# Supplementary material for: The interactome of intact mitochondria by cross-linking mass spectrometry provides evidence for coexisting respiratory supercomplexes
Source: Mol Cell Proteomics. 2017 Dec 8;17(2):216–32. doi: 10.1074/mcp.RA117.000470 (PMC5795388; doi:10.1074/mcp.RA117.000470)
Supplement: Supplemental Data [file supp_RA117.000470_133922_0_supp_23978_fzffwf.zip › spectra_annotation/mito_spectra_annotation/223-1-8-1-7-1.pdf]

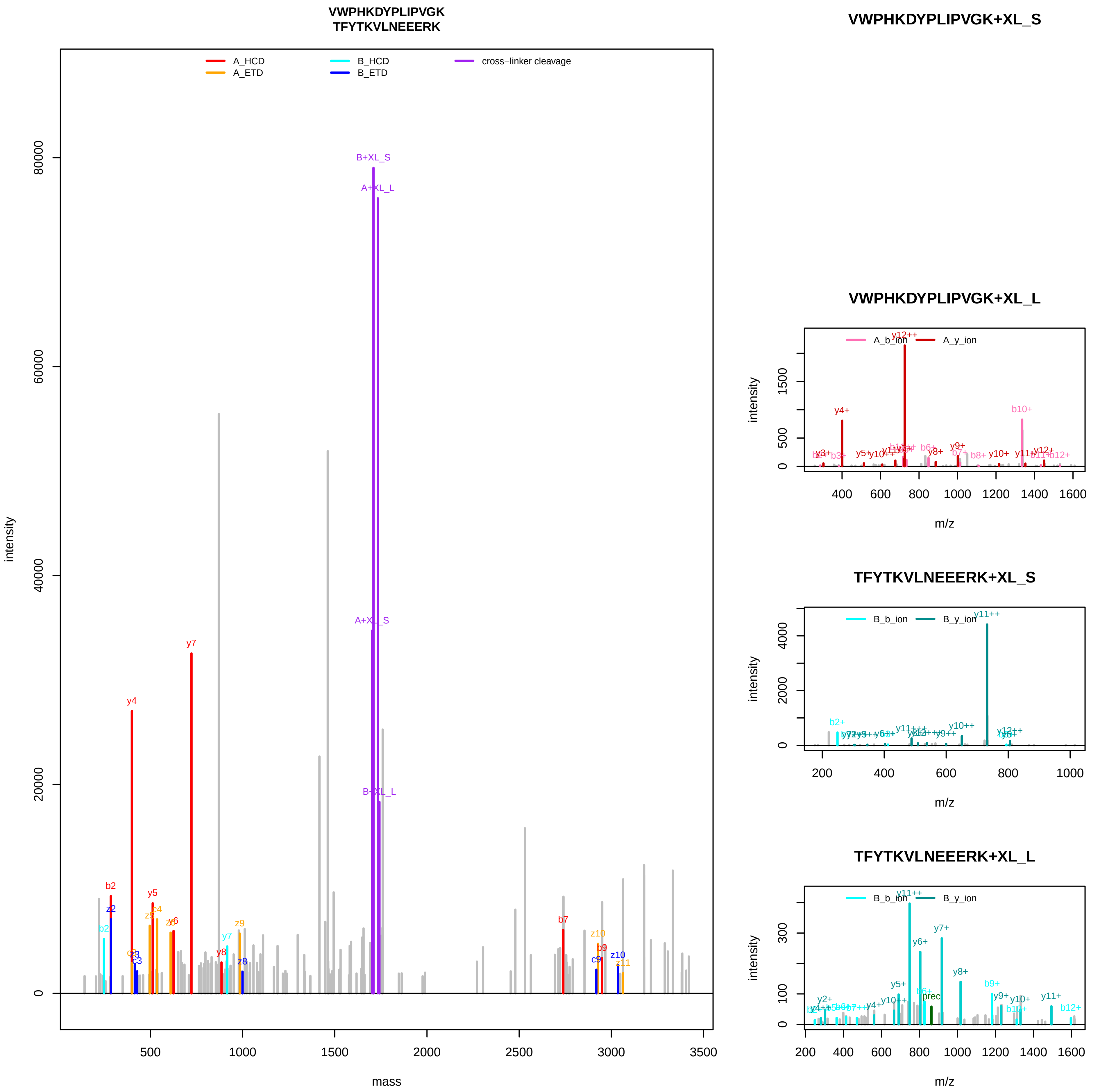
VWPHKDYPLIPVGK
TFYTKVLNEEERK
A_HCD
A_ETD
B_HCD
B_ETD
cross−linker cleavage
B+XL_S
A+XL_L
A+XL_S
B+XL_L
y4
y7
b2
z2
b2
y5
c4
z5
y6
c3
z3
c3
y8
y7
z9
z8
b7
z10
b9
c9
z10
z11
intensity
mass
0
20000
40000
60000
80000
500
1000
1500
2000
2500
3000
3500
VWPHKDYPLIPVGK+XL_S
VWPHKDYPLIPVGK+XL_L
A_b_ion
A_y_ion
y12++
y4+
b10+
y9+
y8+
b6+
b8+
y10+
y12+
y5+
y3+
b3+
b7+
b12+
intensity
m/z
0
500
1500
400
600
800
1000
1200
1400
1600
TFYTKVLNEEERK+XL_S
B_b_ion
B_y_ion
y11++
b2+
y10++
y12++
y9++
intensity
m/z
0
2000
4000
200
400
600
800
1000
TFYTKVLNEEERK+XL_L
B_b_ion
B_y_ion
y11++
y7+
y6+
y8+
y5+
b9+
y9+
y10+
y11+
b12+
y2+
y4+
prec
intensity
m/z
0
100
300
200
400
600
800
1000
1200
1400
1600
